# Isolation of a potentially arsenic-resistant *Halomonas elongata* strain (ml10562) from hypersaline systems in the Peruvian Andes, Cusco

**DOI:** 10.1371/journal.pone.0320639

**Published:** 2025-04-16

**Authors:** Shirly Pila-Lacuta, David Pauccar, Jorge Rojas-Vargas, Ulises E. Rodríguez-Cruz, José Luis Sierra, Hugo G. Castelán-Sánchez, María Antonieta Quispe-Ricalde

**Affiliations:** 1 Departamento de Biología, Facultad de Ciencias Biológicas, Universidad Nacional de San Antonio Abad del Cusco, Cusco, Perú,; 2 Department of Biology, University of Western Ontario, London, Ontario, Canada; 3 Department of Microbiology & Immunology, Schulich School of Medicine & Dentistry, University of Western Ontario, London, Ontario, Canada; 4 Departamento de Ecología Evolutiva, Instituto de Ecología, Universidad Nacional Autónoma de México, México City, Mexico; 5 Laboratorio de Genética y Biotecnología Microbiana, Facultad de Ciencias Biológicas, Universidad Nacional de San Antonio Abad del Cusco, Cusco, Perú; 6 Department of Pathology and Laboratory Medicine, University of Western Ontario, London, Ontario, Canada; Graphic Era Institute of Technology: Graphic Era Deemed to be University, India

## Abstract

*Halomonas elongata* strain ml10562, was isolated from hypersaline that was collected from Acos Peru. Average Nucleotide Identity (ANI) and dDDH (digital DNA-DNA Hybridization) values between strain ml10562 and type strains of *Halomonas elongata* species were 71.0–78.4% and 18.8–21.5%, respectively. The draft genome, spanning 4,075,440 base pairs, has a GC content of 64.2% and contains 3,912 genes. Functional characterization revealed the strain’s ability to tolerate and resist increasing concentrations of sodium arsenate, with a minimum inhibitory concentration of 25 mM. Bioinformatic analysis revealed the presence of two operons, *arsR-arsH-arsB* and *arsJ-gapdh-arsC*, in the genome of strain ml10562, which could play a crucial role in arsenic resistance through transporter-mediated mechanisms. Overall, these results emphasize the potential adaptability of *H. elongata* ml10562 to arsenic-containing environments and extend our understanding of bacterial arsenic resistance mechanisms, allowing promising applications in bioremediation.

## Introduction

The genus *Halomonas* is a group of Gram-negative bacteria with 180 recognized species [1]. Most species are widely distributed in saline habitats such as salt lakes, marine environments, and saline sands as well as soils [2]. It is one of the most widespread groups of halophilic microorganisms found in different geographical locations, perhaps because of its great metabolic and physiological versatility [3–5].

Within this genus, *Halomonas elongata* is an aerobic and moderately halophilic bacillus that belongs to the Halomonadaceae familiy. *H. elongata* thrives in environments with high salt concentrations [2,6]. The main mechanism by which *H. elongata* can grow in high-salt environments is the accumulation of the compatible solvent ectoin. Ectoine can be produced by bacteria either through de novo synthesis from aspartate or by uptake from the environment via the ectoine-specific, osmoregulated TeaABC (transmembrane ectoine ABC) transporter. This transporter system comprises three genes that encode distinct proteins: a large transmembrane protein (TeaC), a small transmembrane protein (TeaB), and a periplasmic substrate-binding protein (TeaA). The TeaA component is part of the tripartite ATP-independent periplasmic (TRAP) transporter family, specialized for ectoine transport in response to osmotic conditions [7–9].

*Halomonas* species, including *H. elongata*, have been found to have the ability to tolerate and even accumulate heavy metals such as arsenic (As) and lead (Pb) in their cells [5,10]. Some arsenic-resistance genes have been described, and their behavior in arsenic-contaminated media has also been reported [4,11,12]. Thus, this species could be a candidate for decontamination of arsenic pollution [12], which has adverse effects on human health [13].

The ability to tolerate and accumulate heavy metals is of great interest for environmental bioremediation [14–16]. *H. elongata* could potentially be used to remove heavy metals from contaminated soils and waters, thereby reducing the negative impact of such pollutants on the environment. In Peru, this is important because various mineral-rich areas suffer from significant arsenic contamination in their natural groundwater and surface drinking water, which can stem from both volcanic activity (natural pollution) and human-induced mining operations (anthropogenic pollution) [17].

The genus *Halomonas* possesses the arsenic resistance operon (*ars* operon), consisting of the genes *arsR*, *arsB*, and *arsC* (collectively known as *arsRBC*), which work together to reduce arsenate and expel it from the cell. More complex operons, such as *arsRDABC* which contains five genes *arsR*, *arsD*, *arsA*, *arsB*, *arsC*., have also been identified in other bacterial strains [18]. Recent studies have further illuminated a novel pathway for arsenate detoxification involving glyceraldehyde-3-phosphate dehydrogenase (GAPDH) and ArsJ, an organoarsenical efflux permease. GAPDH, traditionally known for its role in glycolysis, has been shown to interact synergistically with ArsJ to facilitate the efflux of organoarsenicals from the cell. This discovery suggests a broader role for GAPDH in arsenic resistance beyond its metabolic functions [14,19–22].

The *arsR* gene produces a regulatory protein that binds to arsenate and activates the expression of the operon when the arsenate concentration is high. The *arsD* gene encodes a trans-acting repressor of the arsenical operon. The *arsA* gene codes for an intracellular ATPase protein associated with the arsenic efflux pump encoded by the *arsB* gene. Additionally, the *arsC* gene encodes a protein with reductase activity, which converts arsenate into arsenite, released into the external environment through the arsenic efflux pump. Some bacteria contain the *arsH* gene instead of the *arsC* gene, which also encodes for an arsenate reductase.

However, the *arsRDABC* operon is not the sole mechanism conferring resistance to arsenic. In the case of *Halomonas* sp. strain GFAJ-1, its genome harbors two additional genes: *gapdh* and *arsJ*. These genes encode the enzymes GAPDH (glyceraldehyde 3-phosphate dehydrogenase) and a novel MFS transporter, ArsJ, respectively. Both genes suggested a new pathway for arsenic resistance (Wu et al., 2018).

In the present work, we report the isolation and characterization of *H. elongata* strain ml10562 from hypersaline water at high altitude in Cusco, Peru. Phylogenetic analyses and taxonomic criteria confirmed that strain ml10562 belongs to the species *H. elongata*. Given the extreme environmental conditions in its habitat, we hypothesized that strain ml10562 possesses genetic traits that contribute to arsenic resistance. To confirm this, we performed phenotypic tests to assess its tolerance to arsenite (As III) and arsenate (As V), and genomic analyses to identify potential determinants of arsenic resistance. While arsenite (As III) is generally considered more toxic due to its higher reactivity with cellular components, arsenate (As V) is more stable under oxygen-rich conditions in the environment and remains a significant contaminant in drinking water sources, posing significant risks to ecosystems and human health [23]. Our results show that strain ml10562 is tolerant to elevated concentrations of both arsenite and arsenate, highlighting its adaptability to arsenic-contaminated environments. Genomic analysis revealed two arsenic resistance operons, *arsR-arsH-arsB* and *arsJ-gapdh-arsC*, which may be crucial for arsenic detoxification through transporter-mediated mechanisms. These results suggest that strain ml10562 could be a valuable candidate for bioremediation in arsenic-contaminated waters and soils.

## Materials and methods

### Bacterial strains and culture conditions

Strain ml10562 was isolated from a hypersaline spring, located at 13°57′5″S, 71°44′18″W, 3,088 m elevation, in the Acos district, Acomayo province, Cusco region of Perú. We collected freshwater samples in Acos, an area that is not designated as a protected natural zone by the “Servicio Nacional de Áreas Naturales Protegidas” (Sernanp). The site is publicly accessible, and there are no specific laws or regulations in Peru that restrict sampling in this location. Twenty liters of water were taken from the natural environment, filtered through 0.22 um, and cultured in modified Sea water (SW) agar [Sodium chloride (NaCl) 25%, Magnesium chloride (MgCl_2_) 0.5%, Magnesium sulfate (MgSO_4_) 0.583%, Potassium chloride (KCl) 0.117%, yeast extract 0.05%, Sodium bicarbonate (NaHCO_3_) 0.003%, Calcium chloride (CaCl_2_) 0.0083%, pH:8.0] at 37 °C for seven days. Morphologically-distinct colonies were observed, all of which were newly streaked in SW plates for purification [24].

### Genome sequencing and control quality

Total genomic DNA was extracted from the strain ml10562, which was cultured in SW medium, using the GenElute kit from Sigma-Aldrich (St. Louis, MO, United States). Sequencing reactions were performed using the Illumina MiSeq platform and the Nextera XT DNA library kit, following the protocol (San Diego, CA, United States). Raw sequences were made available in the SRA database under accession number SRR20740642.

The quality of reads were evaluated using FASTQC [25], and the raw sequences were trimmed with a quality ≥Q29 using TrimGalore [26]. Sequence duplicates were removed using CD-HIT-dup [27], and the reads were *de novo* assembly with SPAdes v3.13.0 [28]. Finally, the assembly quality was evaluated with CheckM v1.2.2 [29] with the “genus *Halomonas*” option. The bacterial genome was annotated using RAST tool kit [30]. The genome has been deposited into NCBI under accession number ASM2450512v2.

### Genome assembly, identification and annotation

To conduct the phylogenetic analysis and taxonomic identification, we retrieved twenty-six genomes of the *Halomonas* genus from the NCBI portal (accessed on March 2, 2024). Eight genomes of *H. elongata* were available in the NCBI database, and based on the results of GTDB-Tk v2.0.0, we also included the 18 genomes most closely related to our strain and identified in the NCBI database [31]. In addition, the genomes of *Chromohalobacter canadensis* strain DSM 6769 and *Chromohalobacter sarecensis* strain DSM 15547 were included as outgroups in the phylogenetic tree.

For the phylogenetic reconstruction, we used Proteinortho v6.0.3 to identify the core genome of the twenty-nine set of genomes, using the -identity=50 option [32]. To the core-genome was defined as the single copy orthologous gene families shared by all the genomes. Amino acid sequences were aligned with MAFFT v7 [33], and all positions in the alignment with gaps in 5% or more of the sequences were removed using trimAl v1.4.rev22 [34]. A maximum-likelihood (ML) tree was estimated with IQ-TREE v2.1.2 [35] based on a total of 1,000 bootstrap replicates, using the LG+F+R5 model suggested by the IQ-TREE program.

For taxonomic identification, a whole genome comparison was performed, between our genome and the genome of the type strain *H. elongata* DSM strain 2581, using PyANI [36] and TYGS [37]. PyANI analysis calculates the average nucleotide identity based on MUMmer (ANIm > 95% for species delineation), and TYGS determines the digital DNA-DNA hybridization (dDDH > 70% for species delineation). The genome annotation of the genome of *H. elongata* strain ml10562 was made with prokka v1.14.6 [38], following the default parameters.

### Identification of genes related to resistance to Arsenic

We also performed genome mining on the selected genomes to identify operons associated with arsenic resistance. Specifically, we targeted the *arsR-arsH-arsB* and *arsJ-gapdh-arsC* operons. To achieve this, we conducted an iterative search using PSI–BLAST on the annotated amino acid sequences, applying a cut-off threshold of E < 0.01 to include relevant sequences. The resulting sequences were mapped onto a pre-constructed phylogenetic tree and visualized using ITOL [39].

### Growth in arsenic *H. elongata* ml10562

*H. elongata* strain ml10562 was cultured in a modified SW medium containing 5% NaCl and pH 7.8 ± 0.2, to which sodium arsenate (Na_3_AsO_4_) was added at the following concentrations: 5 mM, 10 mM, 15 mM, 20 mM, 25 mM, 30 mM, 35 mM, and 45 mM. Incubation was performed at 30 °C for 72 hours at 80 rpm, with absorbance measurements taken every 8 hours at a wavelength of 600 nm. The minimal inhibitory concentration (MIC) of sodium arsenate of the strain was the lowest concentration that inhibited their growth. A culture without sodium arsenate was used as a negative control (S1 Table).

## Results

### Genome characterization and arsenic-related genes in *Halomonas* sp. strain ml10562

Strain ml10562 was assembled into 683 contigs with a genome size of 4,075,440 bp and a GC content of 63.25% from 329,142 reads, with a total of 77,677,512 bases sequenced, resulting in a coverage of 10x with an N50 value of 9,225 bp. The genome size and the GC content are similar to the values of the genome of the type strain *H. elongata* strain DSM 2581 (Table 1). The assembly has 95.58% completeness, and 6.77% contamination, according to the CheckM tool analysis. A total of 4,273 coding genes were predicted, representing an increase of 11% compared to the number of coding genes found in the type strain of *Halomonas* and the genome has been deposited into NCBI under accession number ASM2450512v2*.*

**Table 1:** Comparison between the ml10562 genome and the type strain DSM 2581.

Statistics	*H. elongata* ml10562	*H. elongata* DSM 2581
Genome size (bp)	4,075,440	4,061,825
Contigs	683	1
GC (%)	63.50	63.61
N50	9,225	4,061,825
L50	134	1
CDS	4,273	3,841
tRNA	51	68
rRNA	3	12
GenBank Accession number	GCA_024505125.2	GCA_000196875.2

**Fig 1:**
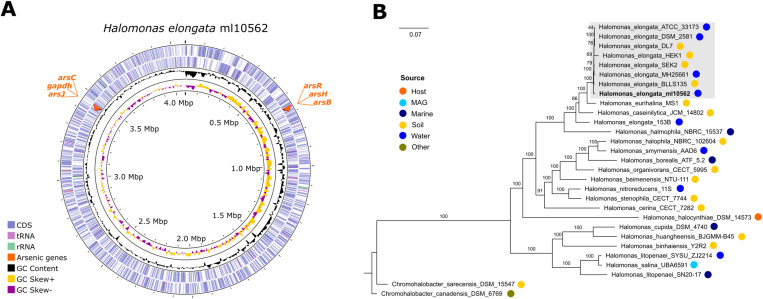
Genome of *H. elongata* strain ml10562 and phylogeny of single copy orthologous gene families. **A.** Circular plot of the genome. Reading from the center outwards, genome size, GC skew, GC content, CDS reverse, and CDS forward. The two arsenic-resistance operons are in red arrows, indicating their synteny. **B.** ML tree of the core genome identified by Proteinortho. The tree was obtained by IQ-TREE with 1,000 bootstrapping. The bacterial genomes were recovered from the NCBI portal (March 2, 2024).

Genome annotation reveals the presence of genes critical for arsenic resistance in two operons (Fig 1A). The first contains the *arsR-arsH-arsB* genes, encoding for the regulator ArsR, the arsenical resistance reductase protein ArsH, and the arsenic transporter ArsB. The second harboring the *arsJ-gapdh-arsC* genes, encoding the MFS transporter ArsJ, the ArsJ-associated glyceraldehyde-3-phosphate dehydrogenase, and the arsenate reductase ArsC.

### Phylogenetic classification analysis

The phylogenetic tree of *H. elongata* strain ml10562 (Fig 1B) was derived from different species of the genus *Halomonas* using the 842 single copy gene families identified, representing the core genome of the used genomes set. The tree was reconstructed with the LG+F+R5 model suggested by the IQ-TREE program. It shows that the closest relatives to ml10562 belong to *H. elongata* species, isolates obtained from soil/water saltern samples. These species form a monophyletic clade, that is well-supported and independent of other *Halomonas* species. *H. elongata* strain 153B falls outside the *H. elongata* species clade, consistent with its NCBI “taxonomy inconclusive” status, suggesting it may belong to a novel *Halomonas* species.

To confirm whether the isolated bacteria was *H. elongata*, ANI and dDDH analyses were performed against the *H. elongata* type strain DSM 2581 genome. ANIm value of 98.47% and dDDH value of 84.80%, above the thresholds for species delineation (ANIm>95%, dDDH>70%), confirm that our strain belongs to *H. elongata* species*.* In conclusion, the phylogenetic analysis and ANI/dDDH values strongly support the identification of the isolated strain ml10562 as *H. elongata*.

### Identification of *ars* operons in *Halomonas* genomes

We used genome mining to identify genes responsible for arsenic resistance encoded by operons in 29 bacterial genomes. Our analysis focused on seven proteins that are encoded by the genes: *arsR, arsH, arsB, arsK, arsJ, gapdh*, and *arsC*. The first operon includes *arsR, arsH*, and *arsB*, while the second operon includes *arsK*, *arsJ*, *gapdh*, and *arsC* [20].

**Fig 2:**
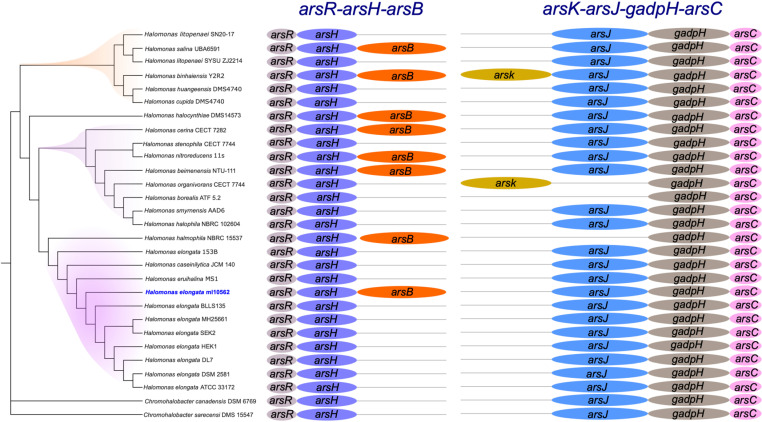
Distribution of operons Ars in *H. elongata* genomes. *Halomonas elongata* ml10562 strain have present two operons, *arsR-arsH-arsB,* and *arsJ-gapdh-arsC*.

Our results show that the proteins encoded by the *arsR* and *arsH* genes are ubiquitous and present in all genomes analyzed. However, the ArsB protein, a transmembrane As(III) efflux permease responsible for the export of arsenite from the cytosol, was identified in only 7 of the 29 genomes. Remarkably, the *H. elongata* ml10562 strain contains all three proteins in the first operon (Fig 2). The ArsR protein functions as an arsenite-responsive repressor that regulates transcription of the *ars* operon. The ArsH protein functions as a reductase with various substrates, including arsenate, which contribute to arsenic resistance by converting arsenate to arsenite and aiding in its subsequent detoxification [20]. The absence of the ArsB protein in many genomes suggests alternative mechanisms for arsenic efflux in these bacteria.

The ArsK protein, which is involved as an arsenic transport protein in the detoxification of arsenic [40], was identified in the genomes of *Halomonas binhaiensis* Y2R2 and *Halomonas organivorans* CECT 5995 [41]. This suggests that these bacteria may have additional arsenic resistance mechanisms beyond the *arsJ-gapdh-arsC* operon. The *arsJ-gapdh-arsC* operon associated with arsenic resistance was almost completely present in all genomes analyzed, except for *Halomonas organivorans* CECT 5995 and *Halomonas halophila* NBRC 102604.

These results suggest a diversity of genetic strategies for arsenic resistance within the genus *Halomonas*, with some strains having a full complement of arsenic resistance proteins, while others rely on alternative mechanisms.

### Arsenite operon genes in arsenic-resistant ml10562 strain

Primers targeting to arsenite transporter gene (*arsB*) successfully amplified a single amplicon of the expected size (approximately 800 pb) and the sequences showed 73% - 80.5% nucleotide sequence identity to putative arsenite efflux pump of *Pseudomonas* sp. and *Halomonas* sp. The length of *arsB* reported was approximate 750 pb, in case of *H. elongata* ml10562 the sequence was 806 pb, and also was similar to the others *arsB* well characterized bacteria sequences with PCR product about 850 pb [42].

The fragment of arsenate reductase (*arsC*), a member of the second operon (*arsC-gapdh-arsJ*), was amplified by new design primers, and the amplicon was 86 pb. Sequence analysis revealed 87.5% similarity to the arsenate reductase (glutaredoxin) of *Onishia taeanensis* strain USBA-857 (WP_112055332.1), previously classified within the genus *Halomonas* [43]. This similarity suggests that strain ml10562 contains this reductase gene. The nucleotide sequences obtained in this study were deposited in the GenBank database with number of access PQ331186.

### *H. elongata* ml10562 has the ability to grow and tolerate different concentrations of sodium arsenate

**Fig 3:**
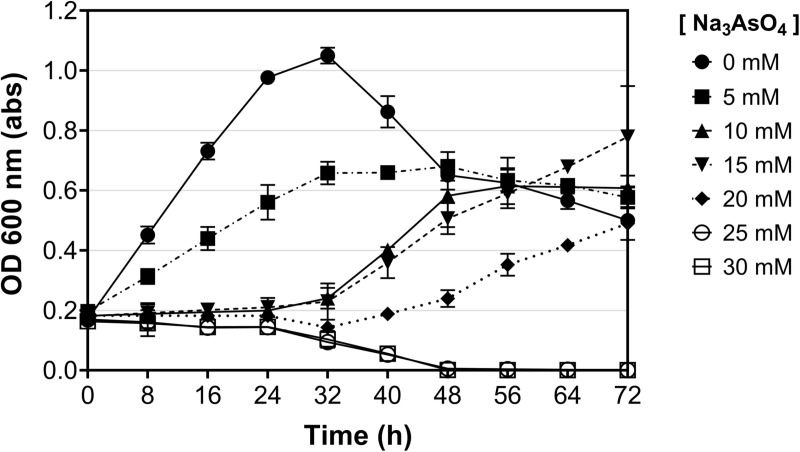
Growth of *H. elongata* strain ml10562 at different concentrations of sodium arsenato. Strain ml10562 had growth from 8 h with 5 mM, while 10 mM and 15 mM had exponential growth after 32 hrs.

*H. elongata* strain ml10562 grown in broth at different concentrations of sodium arsenate during different time periods, and the curves with higher absorbance values are seen at decreasing concentrations of 5 > 10 > 15 > 20 mM Na_3_AsO_4_, clearly showing that tolerance to this compound decreases as the concentration to which this strain is exposed increases (Fig 3, S1 Fig).

The 25 mM and 30 mM curves show a die-off phase, as evidenced by decreased absorbance values. This suggests that the minimum inhibitory concentration of sodium arsenate is 25 mM, as this is the lowest concentration that prevents bacterial growth.

The results show that the strain ml10562 tolerates and is resistant to increasing concentrations of sodium arsenate (Na_3_AsO_4_). As the sodium arsenate concentration increased, a decline in growth was observed, with higher absorbance values corresponding to lower concentrations. Upon gradually increasing the concentration of sodium arsenate, it was observed that at 25 mM, strain ml10562 tolerated the arsenate without any increase in OD for the first 24 hours. After 48 hours, the OD values declined close to zero (OD < 0.0056).

## Discussion

Arsenic contamination represents a severe ecological and health hazard, exacerbated by industrial activities such as mining that release arsenic into water and soil. Bacteria play a crucial role in the biogeochemical cycling of arsenic, acting as both mobilizers and sinks in these contaminated ecosystems. Understanding the genetic basis of arsenic resistance in bacteria provides insights into their potential applications in bioremediation and makes arsenic-tolerant microorganisms valuable resources for the mitigation of arsenic pollution [44,45].

The Peruvian mining sector, particularly in regions such as Cerro de Pasco, has contributed to significant arsenic contamination in interconnected aquifers, some of which include hypersaline and freshwater systems in the Andes [46,47]. Hypersaline environments, often characterized by extreme conditions, are known to harbor a variety of arsenic-resistant bacteria, as has been reported from saline lakes such as Lake Van, Mono Lake, Searles Lake, and Dali Lake [44]. In this study, we isolated *H. elongata* strain ml10562 from a hypersaline region in the Peruvian Andes, an environment in which resistance to heavy metals, including arsenic, could provide a survival advantage. The adaptation of this strain to both hypersaline and arsenic-contaminated conditions suggests its potential as a model organism for understanding arsenic resistance in extreme environments and highlights its potential utility for bioremediation specifically in hypersaline, arsenic-contaminated ecosystems.

Genome sequencing and assembly confirmed that strain ml10562 belongs to *H. elongata*, a species that is well adapted to high salinity and has been shown to exhibit arsenic tolerance. A comparative genomic analysis revealed two arsenic resistance operons: *arsR-arsH-arsB* and *arsJ-gapdh-arsC*. These operons encode critical functions for arsenic detoxification. The *arsR-arsH-arsB* operon comprises *arsR*, a regulatory gene encoding a repressor that controls expression of the operon in response to arsenic stress [10,11,48,49]; *arsB* (also known as *acr3*), which functions as an efflux pump and facilitates the export of arsenite [As(III)][50]; and *arsH*, which may reduce arsenate to arsenite, providing additional detoxification support [20]. The *arsJ-gapdh-arsC* operon also includes arsenate reductase (*arsC*), which is essential for the conversion of arsenate [As(V)] to arsenite, which can then be actively exported from the cell [19]. The *arsJ* gene, which is also present in other arsenic-resistant *Halomonas* strains, is thought to encode a transporter involved in arsenite export, while *gapdh* may contribute to both arsenic resistance and general cellular metabolism, indicating a dual function [20]. Notably, eight of the twenty-nine *Halomonas* genomes analyzed contain the *arsB* gene for arsenic export, indicating that strains without this gene may rely on alternative efflux mechanisms.

Interestingly, we identified the *arsK* gene in two *Halomonas* genomes. The *arsK* gene encodes an arsenic efflux transporter previously characterized in *Agrobacterium tumefaciens* strain GW4, where it helps to expel arsenic compounds from cells to reduce toxicity [40]. The presence of *arsK* in *Halomonas* suggests possible arsenic resistance mechanisms that should be further investigated to confirm its function and role in detoxification. The absence of *arsK* in strain ml10562 suggests that it relies on alternative arsenic efflux systems or mainly on the *arsJ-gapdh-arsC* operon for arsenic export and detoxification.

Previous studies on arsenic resistance in *Halomonas* species have shown varying degrees of tolerance, suggesting species- or strain-specific adaptations to arsenic. For example, *Halomonas* sp. strain A3H3 showed resistance to 29 mM As(III) and more than 106 mM As(IV) [11], while *Halomonas* sp. strain MG showed a tolerance level of 10.68 mM arsenic [12]. In contrast, strain ml10562 showed moderate tolerance to arsenic, with an MIC at 25 mM sodium arsenate. Although this resistance is lower than that of strain A3H3, it is higher than that of strain MG. These results suggest that strain ml10562 may have adaptations unique to its hypersaline origin, where selection pressure favors intermediate levels of arsenic resistance. In addition, differences in the genetic make-up of arsenic resistance mechanisms, such as variations in operons or the presence or absence of specific transporters, could be responsible for the diversity in arsenic tolerance between *Halomonas* strains.

## Conclusions

In conclusion, the genomic analysis of *H. elongata* strain ml10562 revealed key insights into its arsenic resistance mechanisms and phylogenetic relationships within the *Halomonas* genus. The strain exhibited a high degree of genomic completeness and similarity to other strains of *H. elongata* and phylogenetic analysis confirmed the classification of strain ml10562 as a member of the *H. elongata* species. Furthermore, the strain demonstrated tolerance and resistance to increasing concentrations of sodium arsenate, with the minimum inhibitory concentration determined to be 25 mM. This resistance was supported by the presence of arsenic detoxification genes such as *arsH* and *arsJ*, which play crucial roles in mitigating the cellular accumulation of arsenate.

Overall, these findings highlight the adaptability of *H. elongata* strain ml10562 to arsenic-rich environments and contribute to our understanding of the diverse mechanisms bacteria employ to survive in such challenging conditions.

## Further directions

Our study identified two operons, *arsR-arsH-arsB* and *arsJ-gapdh-arsC*, associated with arsenic resistance in *H. elongata* ml10562. While their presence suggests a potential mechanism for arsenic detoxification, further functional characterization is warranted. Techniques like gene knockout and overexpression could elucidate their specific roles in arsenic resistance. Additionally, qPCR or RNA-seq could be used to quantify the expression of these genes (*arsR*, *arsH*, *arsB*, *arsJ*, *gapdh*, *arsC*) under arsenic exposure. This would confirm if they are upregulated in response to arsenic stress. However, the primary objective of this study was to provide a comprehensive genomic analysis of *H. elongata* ml10562, highlighting its potential for future applications, such as bioremediation of arsenic-contaminated environments.

## Supporting information

S1 Fig Growth of *H. elongata* strain ml10562 at different sodium arsenate concentrations on a logarithmic scale. Strain ml10562 showed growth from 8 hours at 5 mM, while at 10 mM and 15 mM exponential growth was observed after 32 hours. The data are presented on a logarithmic scale to emphasize the differences in growth patterns.
(TIF)

S1 Table Curve of growth of *H. elongata* strain ml10562. (XLS)

## References

[1] Parte AC, Sardà Carbasse J, Meier-Kolthoff JP, Reimer LC, Göker M. List of Prokaryotic names with Standing in Nomenclature (LPSN) moves to the DSMZ. Int J Syst Evol Microbiol. 2020;70(11):5607–12. doi: 10.1099/ijsem.0.004332 32701423 PMC7723251

[2] Poli A, Nicolaus B, Denizci AA, Yavuzturk B, Kazan D. Halomonas smyrnensis sp. nov., a moderately halophilic, exopolysaccharide-producing bacterium. Int J Syst Evol Microbiol. 2013;63(Pt 1):10–8. doi: 10.1099/ijs.0.037036-0 22328606

[3] Castelán-Sánchez HG, Elorrieta P, Romoacca P, Liñan-Torres A, Sierra JL, Vera I, et al. Intermediate-salinity systems at high altitudes in the Peruvian Andes unveil a high diversity and abundance of bacteria and viruses. Genes (Basel). 2019;10(11):891. doi: 10.3390/genes10110891 31694288 PMC6895999

[4] Mamani JI, Pacheco KB, Elorrieta P, Romoacca P, Castelan H, Davila S, et al. Draft genome sequence of halomonas elongata MH25661 isolated from a saline creek in the Andes of Peru. Microbiol Resour Announc. 2019;8(1):e00934-18. doi: 10.1128/MRA.00934-18 30637380 PMC6318351

[5] Gasperotti AF, Studdert CA, Revale S, Herrera Seitz MK. Draft genome sequence of Halomonas sp. KHS3, a polyaromatic hydrocarbon-chemotactic strain. Genome Announc. 2015;3(2):e00020-15. doi: 10.1128/genomeA.00020-15 25767220 PMC4357742

[6] Vreeland R, Litchfield C, Martin E, Elliot E. Halomonas elongata, a new genus and species of extremely salt-tolerant bacteria. International Journal of Systematic Bacteriology. 1980;30:485–95.

[7] Nakayama H, Yoshida K, Ono H, Murooka Y, Shinmyo A. Ectoine, the compatible solute of *Halomonas elongata*, confers hyperosmotic tolerance in cultured tobacco cells. Plant Physiol. 2000;122(4):1239–47. doi: 10.1104/pp.122.4.1239 10759521 PMC58960

[8] Tetsch L, Kunte HJ. The substrate-binding protein TeaA of the osmoregulated ectoine transporter TeaABC from *Halomonas elongata*: purification and characterization of recombinant TeaA. FEMS Microbiol Lett. 2002;211(2):213–8. doi: 10.1111/j.1574-6968.2002.tb11227.x 12076815

[9] Yu J, Wang Z, Wang J, Mohisn A, Liu H, Zhang Y, et al. Physiological metabolic topology analysis of *Halomonas elongata* DSM 2581T in response to sodium chloride stress. Biotechnol Bioeng. 2022;119(12):3509–25. doi: 10.1002/bit.28222 36062959

[10] Lin Y, Fan H, Hao X, Johnstone L, Hu Y, Wei G, et al. Draft genome sequence of Halomonas sp. strain HAL1, a moderately halophilic arsenite-oxidizing bacterium isolated from gold-mine soil. J Bacteriol. 2012;194(1):199–200. doi: 10.1128/JB.06359-11 22156396 PMC3256601

[11] Koechler S, Plewniak F, Barbe V, Battaglia-Brunet F, Jost B, Joulian C, et al. Genome sequence of *Halomonas* sp. strain A3H3, isolated from arsenic-rich marine sediments. Genome Announc. 2013;1(5):e00819-13. doi: 10.1128/genomeA.00819-13 24115546 PMC3795216

[12] Govarthanan M, Shim J, Kim SA, Kamala-Kannan S, Oh B-T. Isolation and characterization of multi-metal-resistant halomonas sp. mg from tamil nadu magnesite ore soil in India. Curr Microbiol. 2015;71(5):618–23. doi: 10.1007/s00284-015-0897-4 26298269

[13] Chung J-Y, Yu S-D, Hong Y-S. Environmental source of arsenic exposure. J Prev Med Public Health. 2014;47(5):253–7. doi: 10.3961/jpmph.14.036 25284196 PMC4186553

[14] Sher S, Rehman A. Use of heavy metals resistant bacteria-a strategy for arsenic bioremediation. Appl Microbiol Biotechnol. 2019;103(15):6007–21. doi: 10.1007/s00253-019-09933-6 31209527

[15] Yamamura S, Amachi S. Microbiology of inorganic arsenic: From metabolism to bioremediation. J Biosci Bioeng. 2014;118(1):1–9. doi: 10.1016/j.jbiosc.2013.12.011 24507904

[16] Irshad S, Xie Z, Mehmood S, Nawaz A, Ditta A, Mahmood Q. Insights into conventional and recent technologies for arsenic bioremediation: a systematic review. Environ Sci Pollut Res Int. 2021;28(15):18870–92. doi: 10.1007/s11356-021-12487-8 33586109

[17] Bolisetty S, Rahimi A, Mezzenga R. Arsenic removal from Peruvian drinking water using milk protein nanofibril–carbon filters: a field study. Environ Sci: Water Res Technol. 2021;7(12):2223–30. doi: 10.1039/d1ew00456e

[18] Shi K, Fan X, Qiao Z, Han Y, McDermott TR, Wang Q, et al. Arsenite oxidation regulator AioR regulates bacterial chemotaxis towards arsenite in *Agrobacterium tumefaciens* GW4. Sci Rep. 2017;7:43252. doi: 10.1038/srep43252 28256605 PMC5335332

[19] Carlin A, Shi W, Dey S, Rosen BP. The ars operon of *Escherichia coli* confers arsenical and antimonial resistance. J Bacteriol. 1995;177(4):981–6. doi: 10.1128/jb.177.4.981-986.1995 7860609 PMC176692

[20] Wu S, Wang L, Gan R, Tong T, Bian H, Li Z, et al. Signature arsenic detoxification pathways in *Halomonas* sp. strain GFAJ-1. mBio. 2018;9(3):e00515-18. doi: 10.1128/mBio.00515-18 29717010 PMC5930303

[21] Zhao F-J. A novel pathway of arsenate detoxification. Mol Microbiol. 2016;100(6):928–30. doi: 10.1111/mmi.13395 27072877

[22] Chen J, Yoshinaga M, Garbinski LD, Rosen BP. Synergistic interaction of glyceraldehydes-3-phosphate dehydrogenase and ArsJ, a novel organoarsenical efflux permease, confers arsenate resistance. Mol Microbiol. 2016;100(6):945–53. doi: 10.1111/mmi.13371 26991003 PMC4992400

[23] Oremland RS, Stolz JF. Arsenic, microbes and contaminated aquifers. Trends Microbiol. 2005;13(2):45–9. doi: 10.1016/j.tim.2004.12.002 15680760

[24] Koblitz J. Sea Water Agar. Available: https://mediadive.dsmz.de/medium/246

[25] Andrews S. FastQC: a quality control tool for high throughput sequence data. 2017.

[26] Krueger F, James F, Ewels P, Afyounian E, Schuster-Boeckler B. TrimGalore: v0.6.7. 2021. doi: 10.5281/zenodo.5127899

[27] Fu L, Niu B, Zhu Z, Wu S, Li W. CD-HIT: accelerated for clustering the next-generation sequencing data. Bioinformatics. 2012;28(23):3150–2. doi: 10.1093/bioinformatics/bts565 23060610 PMC3516142

[28] Bankevich A, Nurk S, Antipov D, Gurevich AA, Dvorkin M, Kulikov AS, et al. SPAdes: a new genome assembly algorithm and its applications to single-cell sequencing. J Comput Biol. 2012;19(5):455–77. doi: 10.1089/cmb.2012.0021 22506599 PMC3342519

[29] Parks DH, Imelfort M, Skennerton CT, Hugenholtz P, Tyson GW. CheckM: assessing the quality of microbial genomes recovered from isolates, single cells, and metagenomes. Genome Res. 2015;25(7):1043–55. doi: 10.1101/gr.186072.114 25977477 PMC4484387

[30] Brettin T, Davis JJ, Disz T, Edwards RA, Gerdes S, Olsen GJ, et al. RASTtk: a modular and extensible implementation of the RAST algorithm for building custom annotation pipelines and annotating batches of genomes. Sci Rep. 2015;5:8365. doi: 10.1038/srep08365 25666585 PMC4322359

[31] Chaumeil P-A, Mussig AJ, Hugenholtz P, Parks DH. GTDB-Tk v2: memory friendly classification with the genome taxonomy database. Bioinformatics. 2022;38(23):5315–6. doi: 10.1093/bioinformatics/btac672 36218463 PMC9710552

[32] Lechner M, Findeiss S, Steiner L, Marz M, Stadler PF, Prohaska SJ. Proteinortho: detection of (co-)orthologs in large-scale analysis. BMC Bioinformatics. 2011;12:124. doi: 10.1186/1471-2105-12-124 21526987 PMC3114741

[33] Katoh K, Rozewicki J, Yamada KD. MAFFT online service: multiple sequence alignment, interactive sequence choice and visualization. Brief Bioinform. 2019;20(4):1160–6. doi: 10.1093/bib/bbx108 28968734 PMC6781576

[34] Capella-Gutiérrez S, Silla-Martínez JM, Gabaldón T. trimAl: a tool for automated alignment trimming in large-scale phylogenetic analyses. Bioinformatics. 2009;25(15):1972–3. doi: 10.1093/bioinformatics/btp348 19505945 PMC2712344

[35] Nguyen L-T, Schmidt HA, von Haeseler A, Minh BQ. IQ-TREE: a fast and effective stochastic algorithm for estimating maximum-likelihood phylogenies. Mol Biol Evol. 2015;32(1):268–74. doi: 10.1093/molbev/msu300 25371430 PMC4271533

[36] Pritchard L, Glover RH, Humphris S, Elphinstone JG, Toth IK. Genomics and taxonomy in diagnostics for food security: soft-rotting enterobacterial plant pathogens. Anal Methods. 2016;8(1):12–24. doi: 10.1039/c5ay02550h

[37] Meier-Kolthoff JP, Göker M. TYGS is an automated high-throughput platform for state-of-the-art genome-based taxonomy. Nat Commun. 2019;10(1):2182. doi: 10.1038/s41467-019-10210-3 31097708 PMC6522516

[38] Seemann T. Prokka: rapid prokaryotic genome annotation. Bioinformatics. 2014;30(14):2068–9. doi: 10.1093/bioinformatics/btu153 24642063

[39] Letunic I, Bork P. Interactive Tree Of Life (iTOL) v5: an online tool for phylogenetic tree display and annotation. Nucleic Acids Res. 2021;49(W1):W293–6. doi: 10.1093/nar/gkab301 33885785 PMC8265157

[40] Shi K, Li C, Rensing C, Dai X, Fan X, Wang G. Efflux transporter ArsK is responsible for bacterial resistance to arsenite, antimonite, trivalent roxarsone, and methylarsenite. Appl Environ Microbiol. 2018;84(24):e01842-18. doi: 10.1128/AEM.01842-18 30315082 PMC6275340

[41] Li X, Lu H, Wang Q, Yang H, Yang H, Wu J, et al. Halomonas binhaiensis sp. nov., isolated from saline-alkali soil. Int J Syst Evol Microbiol. 2022;72(12):10.1099/ijsem.0.005652. doi: 10.1099/ijsem.0.005652 36748689

[42] Achour AR, Bauda P, Billard P. Diversity of arsenite transporter genes from arsenic-resistant soil bacteria. Res Microbiol. 2007;158(2):128–37. doi: 10.1016/j.resmic.2006.11.006 17258434

[43] de la Haba RR, Arahal DR, Sánchez-Porro C, Chuvochina M, Wittouck S, Hugenholtz P, et al. A long-awaited taxogenomic investigation of the family Halomonadaceae. Front Microbiol. 2023;14:1293707. doi: 10.3389/fmicb.2023.1293707 38045027 PMC10690426

[44] Omeroglu E, Sudagidan M, Ogun E. Arsenic pollution and anaerobic arsenic metabolizing bacteria in Lake Van, the world’s largest soda lake. Life. 2022;12:1900. 36431035 10.3390/life12111900 PMC9694729

[45] Sedláček V, Kryl M, Kučera I. The ArsH protein product of the paracoccus denitrificans ars operon has an activity of organoarsenic reductase and is regulated by a redox-responsive repressor. Antioxidants (Basel). 2022;11(5):902. doi: 10.3390/antiox11050902 35624766 PMC9137774

[46] Piñeiro XF, Ave MT, Mallah N, Caamaño-Isorna F, Jiménez ANG, Vieira DN, et al. Heavy metal contamination in Peru: implications on children’s health. Sci Rep. 2021;11(1):22729. doi: 10.1038/s41598-021-02163-9 34815466 PMC8611049

[47] Featured: Cerro de Pasco. [cited 3 Nov 2024]. Available: https://www.source-international.org/featured-cerro-de-pasco

[48] Wang L, Shao Z. Aerobic denitrification and heterotrophic sulfur oxidation in the genus halomonas revealed by six novel species characterizations and genome-based analysis. Front Microbiol. 2021;12:652766. doi: 10.3389/fmicb.2021.652766 33815342 PMC8014003

[49] Ben FI, Zhang C, Li YP, Zhao Y, Alwathnani HA, Saquib Q, et al. Distribution of arsenic resistance genes in Prokaryotes. Front Microbiol. 2018;9: 2473. 30405552 10.3389/fmicb.2018.02473 PMC6205960

[50] Yang H-C, Fu H-L, Lin Y-F, Rosen BP. Pathways of arsenic uptake and efflux. Curr Top Membr. 2012;69:325–58. doi: 10.1016/B978-0-12-394390-3.00012-4 23046656 PMC4578627

