# Supplementary figures and images for: Isolation of a potentially arsenic-resistant Halomonas elongata strain (ml10562) from hypersaline systems in the Peruvian Andes, Cusco
Source: PLoS One. 2025 Apr 16;20(4):e0320639. doi: 10.1371/journal.pone.0320639 (PMC12002479; doi:10.1371/journal.pone.0320639)

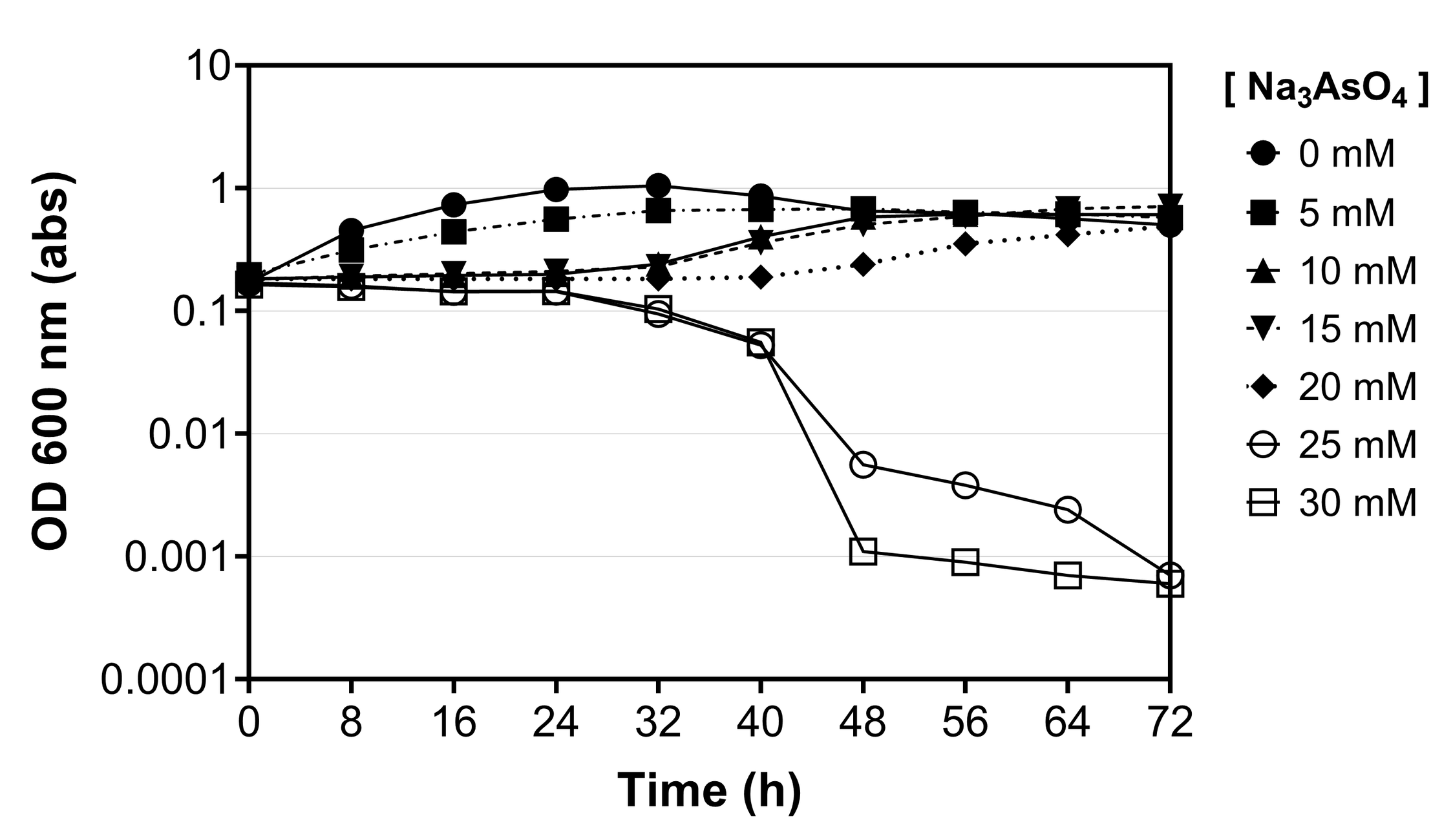

Supplement: S1 Fig — Strain ml10562 showed growth from 8 hours at 5 mM, while at 10 mM and 15 mM exponential growth was observed after 32 hours. The data are presented on a logarithmic scale to emphasize the differences in growth patterns. (TIF) [file pone.0320639.s001.tif]
